# Supplementary material for: Automated PD-L1 Scoring Using Artificial Intelligence in Head and Neck Squamous Cell Carcinoma
Source: Cancers (Basel). 2021 Aug 31;13(17):4409. doi: 10.3390/cancers13174409 (PMC8431396; doi:10.3390/cancers13174409)
Supplement: Supplementary file 1 [file cancers-13-04409-s001.zip › cancers-1313040-supplementary/cancers-1313040-supplementary material final/cancers-1313040-supplementary material 1/cancers-1313040-supplementary-table-figures.pdf]

cancers MDPI

Supplementary Material: Automated PD-L1 Scoring Using Artificial Intelligence in Head and Neck Squamous Cell Carcinoma

Behrus Puladi, Mark Ooms, Svetlana Kintsler, Khosrow Siamak Houschyar, Florian Steib, Ali Modabber, Frank Hölzle, Ruth Knüchel-Clarke and Till Braunschweig

Table S1. Mean cell measurements of tumor, immune and stromal cells. Measured features are based on cell segmentation with StarDist in QuPath. OD: Optical density;.

Feature	Tumor cells	Immune cells	Stromal cells
**Cell: Area µm²**	**165.09**	**89.74**	136.52
Cell: Circularity	0.84	0.80	0.86
Cell: Length µm	48.49	36.93	43.94
Cell: Max diameter µm	17.66	13.63	15.77
Cell: Min diameter µm	12.44	9.34	11.49
Cell: Solidity	0.97	0.97	0.98
Nucleus: Area µm²	47.89	25.90	24.23
Nucleus: Circularity	0.91	0.92	0.88
Nucleus: Length µm	25.09	18.54	18.41
Nucleus: Max diameter µm	9.28	6.61	6.91
Nucleus: Min diameter µm	6.40	5.05	4.56
Nucleus: Solidity	0.99	0.99	0.99
Nucleus/Cell area ratio	0.30	0.31	0.19
Detection probability	0.72	0.68	0.67
Hematoxylin: Cell: Max (OD)	0.75	0.94	0.81
Hematoxylin: Cell: Mean (OD)	0.26	0.33	0.19
Hematoxylin: Cell: Median (OD)	0.22	0.28	0.13
Hematoxylin: Cell: Min (OD)	0.02	0.02	–0.01
Hematoxylin: Cell: Std.Dev. (OD)	0.16	0.21	0.17
Hematoxylin: Cytoplasm: Max (OD)	0.58	0.74	0.57
Hematoxylin: Cytoplasm: Mean (OD)	0.18	0.23	0.13
Hematoxylin: Cytoplasm: Median (OD)	0.16	0.19	0.10
Hematoxylin: Cytoplasm: Min (OD)	0.02	0.02	–0.01
Hematoxylin: Cytoplasm: Std.Dev. (OD)	0.10	0.14	0.09
Hematoxylin: Membrane: Max (OD)	0.48	0.67	0.42
Hematoxylin: Membrane: Mean (OD)	0.18	0.24	0.12
Hematoxylin: Membrane: Median (OD)	0.16	0.19	0.10
Hematoxylin: Membrane: Min (OD)	0.03	0.03	0.00
Hematoxylin: Membrane: Std.Dev. (OD)	0.10	0.16	0.09
Hematoxylin: Nucleus: Max (OD)	0.74	0.92	0.80
Hematoxylin: Nucleus: Mean (OD)	0.43	0.56	0.46
Hematoxylin: Nucleus: Median (OD)	0.43	0.56	0.46
Hematoxylin: Nucleus: Min (OD)	0.16	0.20	0.13
Hematoxylin: Nucleus: Std.Dev. (OD)	0.12	0.16	0.16

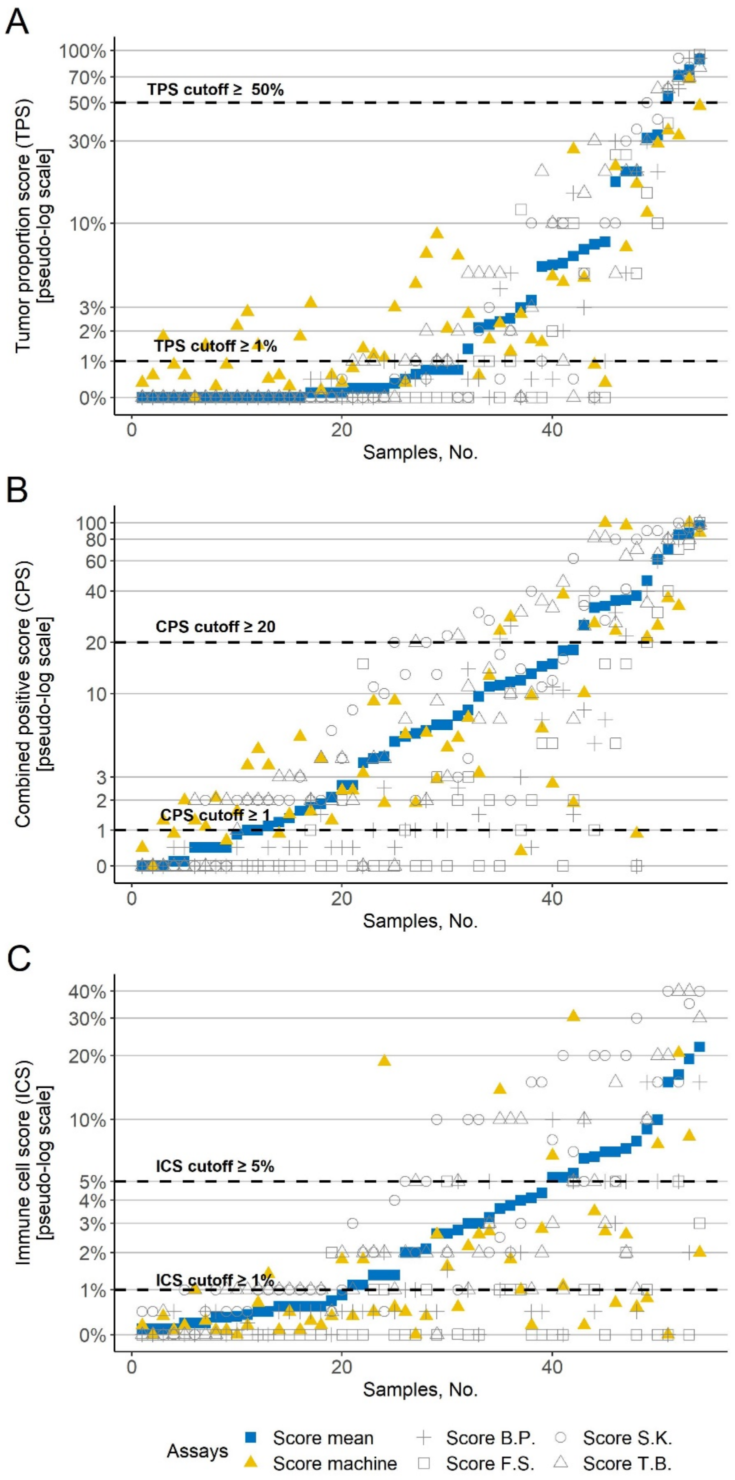

Figure S1. Results of PD-L1 scoring. (**A**-**C**) Three PD-L1 scores for all cases. ■ PD-L1 average score of the four human investigators. ▲Automated PD-L1 score based. The gray symbols correspond to the four investigators (+ B.P., □ F.S., ○ S.K., and △ T.B.); The x-axis shows the samples. The y-axis shows the corresponding PD-L1 scores in pseudo-log scale. The dashed line indicates common cutoffs. The values are sorted in ascending order according to the PD-L1 average score of the four investigators. (**A**) Tumor proportion score (TPS). (**B**) Combined positive score (CPS). (**C**) Immune cell score (ICS).

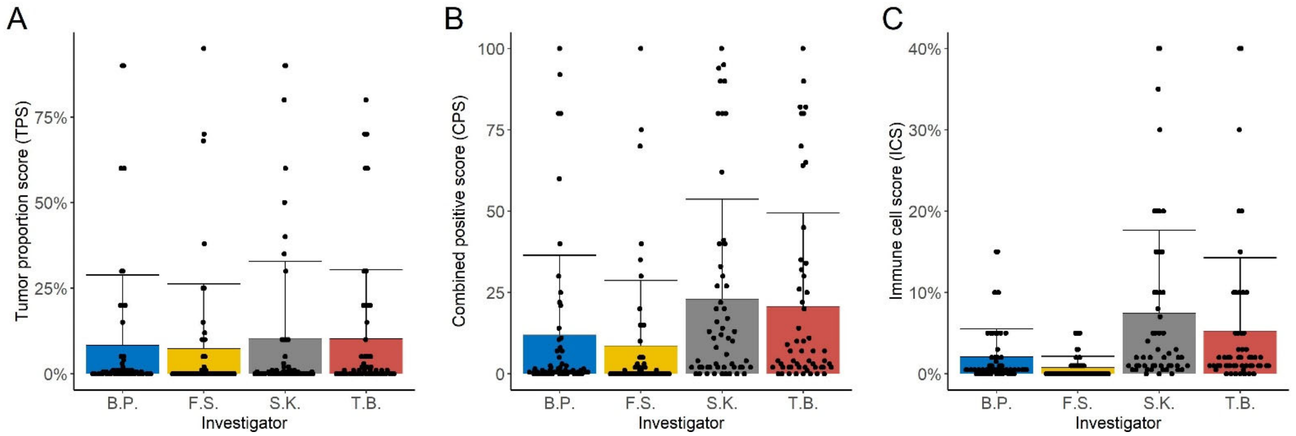

Figure S2. Results of PD-L1 scoring. (**A**-**C**). PD-L1 scores of the four human investigators; The height of the bars corresponds to the mean and the error bar corresponds to the standard deviation of the PD-L1 scores of the corresponding investigator. The black dots correspond to individual measurements. The x-axis shows the investigators. The y-axis shows the corresponding PD-L1 scores. F.S., S.K. and T.B. are specialists in pathology. B.P. also performed scoring after intensive training. The scores of B.P. are in the range of other three investigators. (**A**) Tumor proportion score (TPS). (**B**) Combined positive score (CPS). (**C**) Immune cell score (ICS).
